# Supplementary material for: Lamp1 Deficiency Enhances Sensitivity to α-Synuclein and Oxidative Stress in Drosophila Models of Parkinson Disease
Source: Int J Mol Sci. 2022 Oct 28;23(21):13078. doi: 10.3390/ijms232113078 (PMC9657416; doi:10.3390/ijms232113078)
Supplement: Supplementary file 1 [file ijms-23-13078-s001.zip › ijms-1928284-supplementary.pdf]

Table S1. Genotypes of the *Drosophila* strains used in this study.

w^{1118}/ Y

w^{1118}/ Y; *Lamp1*$^{6.1}$ FRT40A/ *Lamp1*$^{6.1}$ FRT40A

w^{1118}/ Y; *Lamp1*^{11B}/ *Lamp1*^{11B}

w^{1118}/ Y; *Lamp1*$^{6.1}$ FRT40A/ *Lamp1*$^{6.1}$ FRT40A; +/ *UAS-SNCA*^{A30P}

w^{1118}/ Y; *Lamp1*$^{6.1}$ FRT40A/ *Lamp1*$^{6.1}$ FRT40A; *NP6510-Gal4*/+

w^{1118}/ Y; *Lamp1*$^{6.1}$ FRT40A/ *Lamp1*$^{6.1}$ FRT40A; *NP6510-Gal4*/ *UAS-SNCA*^{A30P}

tub-Lamp1/ Y; *Lamp1*$^{6.1}$ FRT40A/ *Lamp1*$^{6.1}$ FRT40A

tub-Lamp1/ Y; *Lamp1*$^{6.1}$ FRT40A/ *Lamp1*$^{6.1}$ FRT40A; +/ *UAS-SNCA*^{A30P}

tub-Lamp1/ Y; *Lamp1*$^{6.1}$ FRT40A/ *Lamp1*$^{6.1}$ FRT40A; *NP6510-Gal4*/ +

tub-Lamp1/ Y; *Lamp1*$^{6.1}$ FRT40A/ *Lamp1*$^{6.1}$ FRT40A; *NP6510-Gal4*/ *UAS-SNCA*^{A30P}

w^{1118}/ Y; +/+; +/ *UAS-SNCA*^{A30P}

w^{1118}/ Y; +/+; *NP6510-Gal4*/ +

w^{1118}/ Y; +/+; *NP6510-Gal4*/ *UAS-SNCA*^{A30P}

w^{1118}/ Y; *Lamp1*^{11B}/ *Lamp1*^{11B}; +/ *UAS-SNCA*^{A30P}

w^{1118}/ Y; *Lamp1*^{11B}/ *Lamp1*^{11B}; *NP6510-Gal4*/ +

w^{1118}/ Y; *Lamp1*^{11B}/ *Lamp1*^{11B}; *NP6510-Gal4*/ *UAS-SNCA*^{A30P}

tub-Lamp1/ Y; *Lamp1*^{11B}/ *Lamp1*^{11B}

tub-Lamp1/ Y; *Lamp1*^{11B}/ *Lamp1*^{11B}; +/ *UAS-SNCA*^{A30P}

tub-Lamp1/ Y; *Lamp1*^{11B}/ *Lamp1*^{11B}; *NP6510-Gal4*/+

tub-Lamp1/ Y; *Lamp1*^{11B}/ *Lamp1*^{11B}; *NP6510-Gal4*/ *UAS-SNCA*^{A30P}

w^{1118}/ Y; *Lamp1*$^{6.1}$ FRT40A/ *Lamp1*$^{6.1}$ FRT40A; *nSyb-Gal4*/+

w^{1118}/ Y; *Lamp1*$^{6.1}$ FRT40A/ *Lamp1*$^{6.1}$ FRT40A; *nSyb-Gal4*/ *UAS-SNCA*^{A30P}

w^{1118}/ Y; *Lamp1*^{11B}/ *Lamp1*^{11B}; *nSyb-Gal4*/ +

w^{1118}/ Y; *Lamp1*^{11B}/ *Lamp1*^{11B}; *nSyb-Gal4*/ *UAS-SNCA*^{A30P}
